# Microwave sensing dataset for noninvasive monitoring of ventricle enlargement due to Alzheimer's disease

**DOI:** 10.1016/j.dib.2023.109006

**Published:** 2023-02-23

**Authors:** Rahmat Ullah, Imran Saied, Tughrul Arslan

**Affiliations:** School of Engineering, University of Edinburgh, Scotland

**Keywords:** Alzheimer's disease, Brain imaging, Microwave sensing data, Lateral ventricle enlargement, Radar data

## Abstract

This paper presents a dataset generated from a comprehensive study on the potential of microwave imaging for early detection or monitoring of different stages of Alzheimer's disease. The study includes collecting and analyzing frequency-domain data using a radar-based head imaging system. The data was obtained from lamb brain phantoms designed to mimic lateral ventricle enlargement, a common symptom of Alzheimer's disease. The article provides detailed descriptions of the data collection method, experimental setup, and different phantoms used. Additionally, the article highlights the importance and potential of the dataset to be used for evaluating and validating new signal processing and imaging techniques. The dataset includes magnitude and phase information for both reflected and transmitted signals making it useful to evaluate radar-based signal processing and imaging techniques. The dataset is open-source and available to the scientific community, providing a valuable resource for researchers to advance their understanding of the potential use of microwave imaging techniques for detecting or monitoring Alzheimer's disease.

**Specifications Table**

**Table:** 

Subject	Biomedical Engineering, Signal Processing, Medical imaging

Specific subject area	Microware Sensing
Type of data	Table (Microsoft Excel Worksheet (.xlsx))
How the data were acquired	The data for this study were acquired using an experimental setup that included a personal computer (PC), a Vector Network Analyzer (VNA) of the Hewlett-Packard (HP) 8753 model, a wearable radio frequency (RF) device, and a skull model containing fabricated phantoms. The VNA was used to generate signals and capture the reflected and transmitted signals from the skull model. The PC was connected to the VNA through a General Purpose Interface Bus (GPIB) to send commands for the VNA to generate signals at various frequencies, with a range from 300 kHz to 3 GHz. The VNA captured the reflected and transmitted data, referred to as S11 and S21, and transferred them to the PC. The wearable RF device was worn on the head model and used to transmit microwave signals into the skull model. The VNA used in the experiments had a dynamic range of up to 100 dB, ensuring the capture of high-quality data. The skull model was designed to mimic lateral ventricle enlargement, a common symptom of Alzheimer's disease (AD).
Data format	Raw (Labeled)
Description of data collection	The data was collected using a skull model containing lamb brains to mimic a human brain. The skull model can be dismantled in order to accommodate the placement of lamb brains and the formation of cavities to represent lateral ventricle enlargement. For this purpose, objects resembling cerebrospinal fluid (CSF) were fabricated and inserted into a cranial cavity. The volumes of the samples used in the experiments were 22.6 mm^3^, 56.5 mm^3^, 113 mm^3^, and 226 mm^3^. The volume refers to the volume of the CSF object used in each experiment. Experiments were performed using unidirectional antennas to collect the reflected and transmitted signals.
Data source location	• Institution: School of Engineering, The University of Edinburgh • City/Town/Region: Edinburgh, United Kingdom
Data accessibility	Repository name: Microwave Sensing Dataset for Noninvasive Monitoring of Ventricle Enlargement due to Alzheimer's Disease Data identification number: 10.17632/y69xddd26p.2. Direct URL to data: https://data.mendeley.com/datasets/y69xddd26p
Related research article	Saied, Imran M., and Tughrul Arslan. “Noninvasive wearable RF device towards monitoring brain atrophy and lateral ventricle enlargement.” IEEE Journal of Electromagnetics, RF and Microwaves in Medicine and Biology 4, no. 1 (2019): 61–68 [1].

## Value of the Data

• Researchers in the fields of radar-based signal processing or imaging can greatly benefit from this dataset, as experimental data is scarce in these fields.
• This experimental dataset has the potential to be used for verification of simulation results.
• The dataset could be used to develop new algorithms for radar-based head imaging systems.
• The data can be transformed to the time domain using techniques such as Fourier transforms in order to be used in time-shift-based processing and imaging techniques. [2].
• The data contains magnitude and phase information for both reflected and transmitted signals, allowing for the investigation of advanced clutter removal techniques.
• The dataset is a valuable resource for researchers working on advancing microwave-based head diagnostics, particularly for detecting or monitoring Alzheimer's disease.

## 1 Objective

AD is becoming one of the world's fastest-growing diseases due to the rapid aging of the population [2]. Therefore, it is of the utmost importance to develop and adopt noninvasive approaches for detecting and monitoring AD. This dataset was created to examine the potential of microwave imaging to detect and monitor subtle changes in the structure and composition of brain tissue due to AD. The data was collected using a microwave imaging system and lamb-brain phantoms mimicking the lateral ventricle enlargement due to AD [1]. The data article adds value to the original publication by describing the data and experimental setup. This clear explanation of the data and experimental setup provides additional insights into the dynamics of change in the brain due to AD. This will help researchers explore the use of novel radar-based sensing and imaging for the detection/monitoring of AD.

## 2 Data Description

Based on the scattered measurements using two antenna, the data file contains RF data collected using a two-port VNA. The VNA measures the scattering parameters (S-parameters). The data includes the frequency, magnitude, and phase of the S_11_ and S_21_ parameters for five brain phantom samples with different volumes of mock objects recorded at discrete frequency points ranging from 0.2 to 3 GHz. The data was collected using two antenna combinations. The data file contains two sheets for each of the five brain phantoms, with one sheet representing the reflected signal S_11_ and sheet two providing the transmitted signal S_21_.

The S_11_ parameter, also known as the reflection coefficient or return loss, is the ratio of the reflected signals to the incident signals. It measures the power reflected to the source and is expressed in dB. The S_21_ parameter, also known as the transmission coefficient, represents the ratio of the transmitted wave to the incident wave at the input port of a two-port system. It is a measure of the power transmitted through the system and is also expressed in dB.p The data is organized in an Excel sheet with two sheets for each of the five brain phantoms. The first sheet provides the reflected signals (S_11_), and the second provides the transmitted signals (S_21_). The magnitude and phase information of the reflected and transmitted signals are expressed in dB and are provided along with the frequency for two antennas. The first column of the Excel sheets represents the frequency (GHz), and the remaining column's name reflects which phantoms were used. For example, the second column is labeled as “No CSF S21”, representing the magnitude of S_21_ data for a healthy brain phantom (No CSF Object), followed by “No CSF Phase” in the third column, and so on. There are a total of five different compositions of brain phantoms, including a healthy case, that was measured and recorded in the data.

## 3 Experimental Design, Materials and Methods

A wearable device has been developed for RF imaging experiments that contain six monopole antennas specifically designed for head imaging [3]. The device has a hat-like shape and can be easily worn by patients, with the antennas arranged around the inner side of an absorber. It is connected to the VNA via wires, and a host PC controls the VNA. The experiments involve placing the wearable device on top of a skull model containing brain phantom samples and using the VNA to generate and receive signals from the antennas, measuring the S_11_ and S_21_ parameters, and recording the data in the PC.

The experimental setup is composed of several components that are essential for the experiments to be successful. The VNA utilised in the experiments was a Hewlett-Packard (HP) 8753 model with a frequency range of 300 kHz to 3 GHz and a dynamic range of up to 100 dB. This allows for a broad range of frequencies to be analyzed and for high precision in the measurements. The wearable device is composed of six hybrid silicone-textile sensors, two flexible switching circuits, a host PC, and a skull model containing brain phantoms that mimic lateral ventricle enlargement in the human brain due to neurodegenerative diseases. The sensors are designed to be flexible and comfortable to wear, while the switching circuits allow for the active element to be selected and the other switches to be turned off. The bio-phantoms that replicated the brain's dielectric properties were fabricated using a method described in [4].

The skull model is a life-size representation of a human skull, and the brain phantoms are created using whole lamb brains and artificial phantoms representing cerebrospinal fluid (CSF). The experiments involve studying lateral ventricle enlargement atrophy by creating a cavity in the lamb brain and inserting a CSF object inside. The experiments and imaging results for brain atrophy, along with the data, can be found in our previous work [5,6]. The lamb brains mimic a healthy human brain, which is around 1/10th the size of a normal lamb brain and has a volume of approximately 1200 cm^3^ [6]. Various brain phantoms were created using lamb brain samples placed in a skull to emulate the lateral ventricle enlargement. To create a 6 mm diameter cavity in the brain, a plastic ball was placed in the center of the skull model. The model was then frozen to keep the brain in place. Afterwards, the plastic ball was removed to create the cavity.

In order to create the artificial CSF objects used to imitate the lateral ventricle enlargement, four different sizes of an artificial phantom were created. These objects were constructed by mixing water, salt, and agar to form high-dielectric objects. This composition was chosen because it closely mimics the electrical properties of real CSF, which is essential for accurately representing the condition. Once the CSF objects were created, they were inserted into the 6-mm diameter cavity in the lamb brain sample inside the skull model. The volumes of these objects were varied, with four different sizes used in the experiment: 22.6 mm^3^, 56.5 mm^3^, 113 mm^3^, and 226 mm^3^. After the CSF objects were inserted into the cavity, the lamb brain sample was allowed to thaw. This was done to permit the brain tissue to conform to the shape of the CSF object and fill in any remaining gaps, mimicking the ventricular enlargement in the human brain.

The experiments were conducted using a two-port VNA, which only allows for the operation of two antennas at a time. As a result, the data collected in the excel sheet pertains to the scattering parameters (S_11_ and S_21_) of only two antennas. The experiments were repeated with varying volumes of mock objects, which were used to emulate different stages of AD. After the data was collected, it was exported to an excel sheet and labeled accordingly.

## Ethics Statement

This study does not involve any in vivo experiments on animals. Therefore, no permissions were needed.

## CRediT authorship contribution statement

**Rahmat Ullah:** Data curation, Writing – original draft, Writing – review & editing, Formal analysis. **Imran Saied:** Conceptualization, Methodology, Data curation, Writing – review & editing, Formal analysis. **Tughrul Arslan:** Formal analysis, Supervision, Project administration.

## Declaration of Competing Interest

The authors declare that they have no known competing financial interests or personal relationships that could have appeared to influence the work reported in this paper.

## Data Availability

Microwave Sensing Dataset for Noninvasive Monitoring of Ventricle Enlargement due to Alzheimer's Disease (Original data) (Mendeley Data).
